# The Diversity of Nutritional Metabolites: Origin, Dissection, and Application in Crop Breeding

**DOI:** 10.3389/fpls.2019.01028

**Published:** 2019-08-16

**Authors:** Chuanying Fang, Jie Luo, Shouchuang Wang

**Affiliations:** ^1^Hainan Key Laboratory for Sustainable Utilization of Tropical Bioresource, College of Tropical Crops, Hainan University, Haikou, China; ^2^National Key Laboratory of Crop Genetic Improvement and National Center of Plant Gene Research (Wuhan), Huazhong Agricultural University, Wuhan, China

**Keywords:** nutritional metabolites, metabolic diversity, crops, genetic bases, breeding

## Abstract

The chemical diversity of plants is very high, and plant-based foods provide almost all the nutrients necessary for human health, either directly or indirectly. With advancements in plant metabolomics studies, the concept of nutritional metabolites has been expanded and updated. Because the concentration of many nutrients is usually low in plant-based foods, especially those from crops, metabolome-assisted breeding techniques using molecular markers associated with the synthesis of nutritional metabolites have been developed and used to improve nutritional quality of crops. Here, we review the origins of the diversity of nutrient metabolites from a genomic perspective and the role of gene duplication and divergence. In addition, we systematically review recent advances in the metabolomic and genetic basis of metabolite production in major crops. With the development of genome sequencing and metabolic detection technologies, multi-omic integrative analysis of genomes, transcriptomes, and metabolomes has greatly facilitated the deciphering of the genetic basis of metabolic pathways and the diversity of nutrient metabolites. Finally, we summarize the application of nutrient diversity in crop breeding and discuss the future development of a viable alternative to metabolome-assisted breeding techniques that can be used to improve crop nutrient quality.

## Introduction

**Figure 1:**
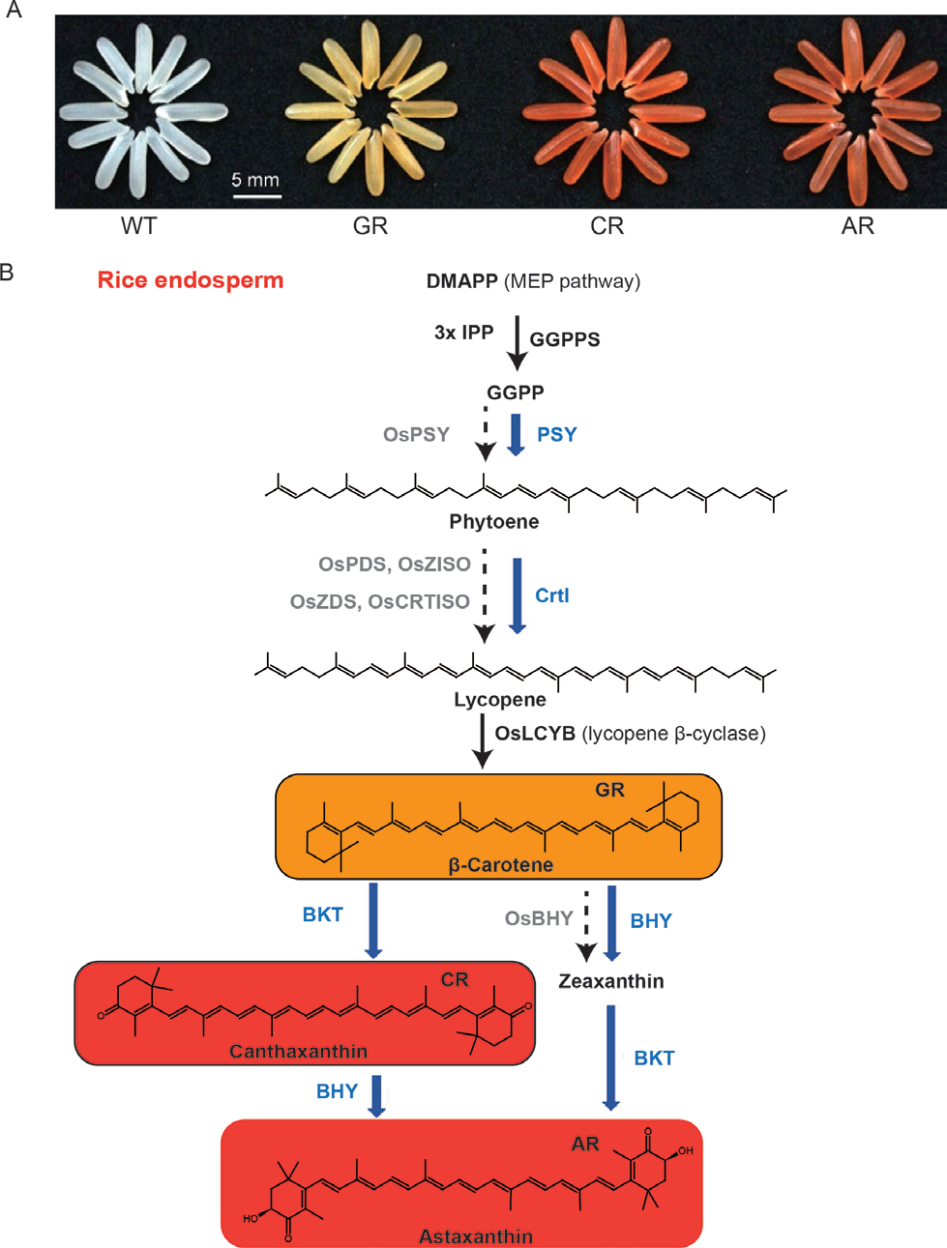
Biofortification of nutrients in rice endosperm. **(A)** Grains of the wild-type (WT), the golden rice (GR), canthaxanthin rice (CR), and astaxanthin rice (AR). **(B)** Simplified carotenoid/canthaxanthin/astaxanthin biosynthesis pathways reconstructed in rice endosperm. The enzymes (in blue) expressed from different combinations of the transgenes, together with those (in black) from the endogenous MEP pathway genes and OsLCYB, catalyze the biosynthesis of β-carotene, canthaxanthin, and astaxanthin as the main products in the GR, CR, and AR lines, respectively. The rate-limiting enzymes of the endogenous genes with no or low levels of expression are shown in gray. The figure is modified with permission from Elsevier.

The nutritional metabolites needed for humans to maintain health are mainly derived from plants, either directly or indirectly when plants are consumed by animals (Dellapenna, 1999). Plant-derived foods, especially crops, provide almost all essential human nutrients such as amino acids, vitamins (tocopherol, ascorbic acid, folic acid), sugars (sucrose, glucose), as well as other health-promoting phytochemicals (Hall et al., 2008). Traditionally, nutritional metabolites are generally not considered to be directly synthesized in the human body, or the specific factors required in their synthetic pathways are lacking or insufficient under certain conditions, and humans must obtain these components from exogenous food (e.g., some amino acids, fatty acids, vitamins) (Hounsome et al., 2008). Golden rice is an important achievement in the improvement of crop nutritional quality through genetically modified technology. Paine et al. developed “Golden rice 2,” introducing *psy* from maize in combination with the *Erwinia uredovora* carotene desaturase (*crtl*) from *Erwinia uredovora* that was used to generate the original golden rice. The β-carotene (provitamin A) content in golden rice is significantly improved, which is helpful in fighting against vitamin A deficiency (Paine et al., 2005). Recently, Zhu et al. introduced four synthetic genes in rice endosperm to achieve astaxanthin biosynthesis, and these four genes are *sZmPSY1*, *sPaCrtl*, *sCrBKT*, and *sHpBHY*, which encode the enzymes phytoene synthase, phytoene desaturase, β-carotene ketolase (BKT), and β-carotene hydroxylase, respectively (**Figure 1**) (Zhu et al., 2018b). However, many other phytochemicals are also nutrients that have a positive effect on human health, such as flavonoids, phytosterols, phenolic acids, carotenoids, polyunsaturated fatty acids, and glucosinolates, which are effective in preventing the occurrence of clinical disease risk (Appleby et al., 2014; Wang et al., 2014). In addition to crop yields and stress resistance, more researches have begun to focus on nutritional quality and how to improve novel nutrients such as anthocyanins, carotenoids, and resveratrol in major crops (Fernie et al., 2006). The anthocyanin content of purple tomato and purple rice has been significantly improved by transgenic technology, and it is considered to be an important manifestation of crop nutrient quality improvement, achieving the goal of crop nutrient biofortification (Butelli et al., 2008; Zhu et al., 2017).

The diversity of nutrients in crops is very complex and difficult to assess. The amount and type of nutritional metabolites are strongly affected by genetic and environmental factors, which are major contributors to nutrient diversity (Hounsome et al., 2008; Ahuja et al., 2010). Because of the inability to move during growth, plants must evolve a series of protective mechanisms to counter the unfavorable environment in order to maintain normal life activities. Environmental factors, including light intensity, temperature, drought, UV radiation, salinity, toxic heavy metals, and so on, induce plants to produce rich metabolic diversity, which provides the possibility of screening and utilization of nutrients in plant (Orcutt and Nilsen, 2000; Hounsome et al., 2008). For example, UV-B radiation induces the accumulation of the multi-functional active flavonoids in the corresponding tissues (Treutter, 2006); under drought conditions, the metabolites, such as carbohydrate metabolites, glycine betaine, proline, ectoine, can be increased to alleviate the damage of plant cells caused by water shortage (Mahajan and Tuteja, 2005). Both biotic and abiotic stresses induce the diversity of metabolites and also affect the nutritional quality of crops. Most metabolites such as sugars, organic acids, amino acids, vitamins, hormones, flavonoids, phenolics, and glucosinolates are essential for plant growth, development, stress adaptation, and defense, and the diversity of these metabolites also determines the nutritional quality, color, taste, and smell as well as antioxidative, anticarcinogenic, anti-inflammatory, antimicrobial, and cholesterol-lowering properties of food (Hounsome et al., 2008). Therefore, deciphering the metabolic diversity and genetic regulation of nutrients makes it possible to develop metabolic markers and genetic loci for metabolome-assisted breeding and biofortification (Luo, 2015; Martin and Li, 2017). Taking advantage of high-throughput metabolic profiling and genome sequencing, a series of advances have been made in the structural identification, biochemical characterization, genetic basis of synthesis, localization, and health benefits of crop nutrient metabolites (Fang et al., 2019).

In the current article, we review the diversity of nutrients in crops, ranging from traditional basic types to the novel types of nutrients that are currently receiving attention. We will summarize the origins of nutrient metabolite diversity from the perspective of the genome, focusing on the essential factors that determine metabolite diversity, including gene duplication and divergence. In addition, we will systematically review the latest advances in studies on the genetic basis of nutrient diversity, especially the forward genetics approach of metabolite-based genome-wide association study (mGWAS), which greatly promotes analyses of the genetic basis of metabolic pathways and diversity. Finally, we will summarize the application of molecular markers related to nutrients and their metabolic biosynthesis in crop breeding and discuss the future impact of metabolome-assisted breeding techniques on crop nutrient quality improvement.

## The Origin of the Diversity of Nutritional Metabolites in the Context of Genome Evolution

Primordial metabolism is generally regarded as chemical intermediates interconnected by a smaller number of ancestral enzymes with multifunctionality (Croteau et al., 2006; Fani and Fondi, 2009). Necessary metabolic processes became established since the appearance of plants in the land (Austin et al., 2004). Currently, plants produce a repository of structurally diverse compounds, including those vital for growth and development and for interactions of plants with environment (Weng et al., 2012). With the development of cross-species metabolic profiling strategies, convergent and divergent evolution of metabolites have been identified in several species (Chen et al., 2016; Tohge et al., 2016; Zhou et al., 2016).

Among the demonstrated mechanisms of the evolution of metabolism, gene duplication and divergence have been documented to be vital sources of the raw material for such evolution (Moghe et al., 2017). There are at least four mechanistic categories of gene duplication, including i) tandem duplication, ii) polyploidy, iii) chromosomal segment duplication, and iv) single-gene transposition–duplication (Freeling, 2009). The cytochrome P450 monooxygenase (P450) family is found to catalyze NADPH- and O_2_-dependent hydroxylation reactions, generally located at the cytoplasmic surface of the endoplasmic reticulum (Werck-Reichhart and Feyereisen, 2000). P450 proteins are involved in the biosynthesis of various primary and secondary metabolites. In plants, P450s are generally categorized into the plant-specific A type and non-plant-specific non-A type, according to the evolutionary relationship (Durst and Nelson, 1995). There were three rounds (i.e., γ, β, and α) of polyploidization in *A. thaliana* and all other Brassicaceae taxa (Bowers et al., 2003). The level of the cytochrome P450 gene evolutionary group was highly enriched throughout evolutionary history. In addition, tandem duplication is also important for the evolution of the cytochrome P450 supergene family (Yu et al., 2017). Furthermore, the evolution of genes encoding homospermidine synthase is also identified to be important for the biosynthesis of pyrrolizidine alkaloid (Ober et al., 2003). Family of plant transcription factors, such as the MADS-box family, and the diterpene synthases, such as isopimaradiene synthase and levopimaradiene/abietadiene synthase, undergo multiple gene duplications that increase secondary metabolites diversity (Keeling et al., 2008; Flagel and Wendel, 2009).

Gene duplications immediately lead to the presence of two identical gene copies. Both copies may remain almost unchanged or diverge functionally. Alternatively, one of the duplicates serves as a pseudogene. To explain the development of new enzymatic functions, at least two hypotheses have been proposed: the neofunctionalization hypothesis and the subfunctionalization hypothesis. According to the first hypothesis, one duplicate’s function resembles that of the ancestral gene, while mutations accumulate in the other gene during evolution, leading to a loss (nonfunctionalization) or a gain (neofunctionalization) of function (Ono, 1973; Rodin and Riggs, 2003). In the latter hypothesis, the functions of the ancestral gene are divided between the daughter genes (Hughes, 1994). Alternatively, duplicated genes may also undergo intraspecific partitioning of functions. Because the divergent evolution of genes involved in plant metabolite synthesis and regulation is too large to be fully reviewed, we will mainly focus on the variation in expression. The subdivision of functions across duplicate genes may manifest as differential expression patterns across multiple genotypes or as differential expression patterns within a single genotype.

Kliebenstein found that duplicated genes have more variable transcript accumulation than the average gene. In addition, the expression of tandem duplicated genes are significantly variable than that of segmentally duplications (Kliebenstein, 2008). Moreover, epigenetic alleles are also critical for the determination of nutritional compounds accumulation.

Vitamin E consisted of tocopherol and its derivates. The first step of tocopherol synthesis is catalyzed by homogentisate phytyl transferase, producing 2-methyl-6-phytylquinol. This precursor is further catalyzed by dimethyl-phytylquinol methyl transferase to synthesize γ- and a-tocopherol (Almeida et al., 2011). Quadrana et al. identified an expression quantitative trait locus (QTL) for vitamin E content in tomato fruits. A retrotransposon was found to be located in the promoter of the methyltransferase-encoded VTE3(1), whose methylation affects the expression of VTE3(1) (Quadrana et al., 2014).

## Dissection of the Genetic Bases of Nutritional Quality in Crops

Plants produce structurally diverse chemicals in order to maintain normal life activities and adapt to ecological environments. Plant metabolites generally consist of primary and secondary metabolites (Luo, 2015). Primary metabolites are thought to be essential for growth and development and play an important role in maintaining the normal life activities of plants, while secondary metabolites are regarded as more closely related to stress responses, helping plants cope with biotic and abiotic stresses in a constantly changing environment (Weng et al., 2012; Wurtzel and Kutchan, 2016). The considerable chemical diversity of plants is the source of nutrients required for human health, and the nutritional status of crops is ultimately dependent on their metabolic composition and content (Memelink, 2005). Metabolomic approaches enable parallel assessment of the levels of a broad range of plant metabolites, providing the possibility to study the diversity of crop nutrient metabolites (Fernie and Schauer, 2009; Samota et al., 2017). The recently developed widely targeted metabolomic approach based on liquid chromatography–mass spectrometry enables high-throughput detection of metabolite content (Chen et al., 2013) and has been used in several species, including rice (Chen et al., 2014; Chen et al., 2016), maize (Wen et al., 2014), citrus (Wang et al., 2016; Wang et al., 2017), and tomato (Zhu et al., 2018a). Comprehensive metabolic profiling and natural variation analysis of flavonoids were carried out in rice, and a total of 91 flavonoids were identified and quantified (Dong et al., 2014). Many advances have been made in the study of plant nutrient biosynthesis, such as vitamin A and oil in maize (Harjes et al., 2008; Li et al., 2013), carotenoids, sugars, and organic acids in tomato (Lu and Li, 2008; Bursac et al., 2017; Tieman et al., 2017), and isoflavones in soybeans (Bursac et al., 2017).

In the process of studying the diversity of crop nutrients, it is critical to clarify how each metabolite is synthesized, transported, and degraded and how the metabolic pathway is regulated (Fang et al., 2019). Advances in different omic technologies, such as genomics, transcriptomics, and metabolomics, have facilitated the qualitative and quantitative analysis of plant metabolites, as well as the detection of candidate genes involved in metabolic synthesis and regulation, which contribute to the diversity of plant metabolite modifications (Oksman-Caldentey and Saito, 2005; Urano et al., 2010; Wang et al., 2019). This strategy to decipher the genetic basis of nutrients is further facilitated by recent advances in next-generation sequencing technology. For example, Sadre et al. recently identified two key genes for camptothecin biosynthesis, namely, TDC1 and TDC2, by analyzing transcriptome and metabolome data. They also found that CYCLASE1 (CYC1) is coexpressed with TDC1, suggesting that it may also be involved in camptothecin biosynthesis (Sadre et al., 2016). Polturak et al. performed multi-species transcriptomic and metabolomic analyses in *Mirabilis jalapa* and additional betalain-producing species to identify candidate genes possibly involved in betalain biosynthesis. Among the identified candidate genes, the betalain-related cytochrome P450 and glucosyltransferase-type genes that catalyze tyrosine hydroxylation and cinnamoyl-glucose formation were further functionally characterized (Polturak and Aharoni, 2018; Polturak et al., 2018). Integration analysis of transcriptome and metabolome data is a powerful tool for deciphering the genetic determinants of metabolic pathways, yet it lacks the ability to unravel the genetic basis of natural variation in the plant metabolome (Fang et al., 2019).

To explore the genetic basis of the crop metabolome, forward genetics based on genomics and metabolomics is being widely used, for example, using the biparental populations to determine QTL mapping and using natural populations for genome-wide association studies (GWASs) (Kliebenstein, 2009; Zhao et al., 2011; Riedelsheimer et al., 2012a; Routaboul et al., 2012; Chen et al., 2014; Chen et al., 2016; Wang et al., 2019). A number of advances have been made in the identification of metabolic quantitative trait locus (mQTL) using ultra-high density maps constructed using next-generation sequencing technologies, for example, integrating ultra-high-density maps of rice and metabolic profiles of seeds for mQTL mapping to analyze the genetic basis of the rice metabolomes and identifying hundreds of mQTLs in flag leaves or germinating seeds (Gong et al., 2013). QTL analysis in tomato seeds revealed colocalization of six amino acids on chromosomes 2, 4, and 10, of which 10 candidate genes related to amino acid metabolism were screened on chromosome 2 (Toubiana et al., 2015). To gain insight into the genetic factors controlling seed metabolism, QTL mapping was performed using the relative content of 311 primary metabolites. A total of 786 mQTLs were unevenly distributed in the genome, forming multiple hotspots. A series of candidate genes, including bZIP10, were identified to provide a basis for further study of the natural variation of Arabidopsis seed metabolism-related genes (Knoch et al., 2017).

Metabolic GWAS (mGWAS), which is used to decipher the genetic basis of plant metabolite biosynthesis and regulation, has made many advances in Arabidopsis (Chan et al., 2010; Wu et al., 2018), maize (Wen et al., 2014; Jin et al., 2017), rice (Luo, 2015; Chen et al., 2016), tomato (Bauchet et al., 2017; Ye et al., 2017), and wheat (Peng et al., 2018). Angelovici et al. performed a non-targeted liquid chromatography–mass spectrometry-based metabolome profiling of 309 Arabidopsis germplasms grown in two separate environments and performed mGWAS analysis to determine 70 significant associations between candidated genes and metabolites (Angelovici et al., 2013). Riedelsheimer et al. performed mGWAS analysis with 56,110 single nucleotide polymorphisms (SNPs) and 118 metabolites in maize inbred lines, identifying 26 different metabolites closely related to maize SNPs, of which *p*-coumaric acid and caffeic acid are closely related to the chromosome 9 region, which contains a gene encoding the key enzyme cinnamoyl-CoA reductase in the synthesis of lignin monomers (Riedelsheimer et al., 2012b). In rice, mGWAS analysis using 175 rice germplasms successfully identified 323 associations between 143 SNPs and 89 secondary metabolites, revealing the genetic mechanism of natural variation in rice secondary metabolite composition (Matsuda et al., 2015). Futhermore, Chen et al. performed quantitative analysis of 840 metabolites on 524 natural rice populations and used mGWAS to identify many important genetic loci associated with different metabolites (Chen et al., 2014). A systematic study of the genetic and biochemical bases of natural variation in flavonoids and polyamines in rice led to the identification of candidate genes related to their biosynthesis by mQTL and mGWAS methods (Peng et al., 2016; Peng et al., 2017). mGWAS can also identify genetic loci that affect most of the target flavor chemicals in tomato. Tieman et al. identified 2,014,488 common SNPs in 398 tomato germplasm genomes and identified 251 flavor-related signals using mGWAS (Tieman et al., 2017). Peng et al., based on six multi-locus GWAS models of 14,646 SNPs, found that 15 candidate genes are involved in free amino acid biosynthesis in wheat and functionally identified the candidate gene *TraesCS1D01G052500* encoding tryptophan decarboxylase, which provides new insights into understanding the biosynthesis of free amino acid in wheat (Peng et al., 2018).

Multi-omics integration analysis and multiple stages of development and different organizational analyses have been increasingly used to provide insight into biological mechanisms since combining multiple different types of datasets can compensate for missing or unreliable information in any single data type (Fang et al., 2019). Metabolic profiling combined with transcriptome analysis has been used to identify new gene clusters and GAME9 transcription factors involved in steroidal glycoalkaloid biosynthesis (Itkin et al., 2013; Cardenas et al., 2016). Joint metabolomic and genomic data subsequently allowed comprehensive refinement of steroidal glycoalkaloid biosynthesis (Schwahn et al., 2014). We recently performed multi-omics analysis of 610 tomato varieties, including genomes, transcriptomes, and metabolomes, to explore changes in fruit metabolomes during human-directed breeding (Zhu et al., 2018a). A total of 13,361 triple relationships (metabolite–SNP–gene), including 371 metabolites, 970 SNPs, and 535 genes, were constructed by mGWAS and eQTL analysis, which facilitated the identification of candidate genes and the clarification of metabolic pathways. For example, the SNP of *SlMYB12* (SNP*y*) discovered by excavating the abovementioned triple relationships was correlated with 69 metabolites and 69 genes in the mGWAS and eQTL analysis, and mutation of the SNP resulted in a decrease in nutrient flavonoid content, resulting in the formation of pink tomato (Zhu et al., 2018a). Multi-omics integrative analysis has also made breakthroughs in the study of tomato flavor and cucumber bitterness. Thirty-seven metabolites, including total soluble solids, glucose, fructose, citric acid, and malic acids, were found to affect tomato flavor, and a total of 251 association signals were detected for 20 traits, including four nonvolatile and 15 volatile flavor chemicals (Tieman et al., 2017). Shang et al. discovered that two TFs regulate nine genes in the cucurbitacin C biosynthetic pathway and proposed a model for how extremely bitter wild cucumber was domesticated into nonbitter cultivars (Shang et al., 2014). These examples show that exploring the biochemical and genetic bases of nutrient diversity can provide new opportunities to increase the level of nutrient biofortification or to change the flavor characteristics that are beneficial to human health (Dixon, 1999).

## The Application of Metabolic Diversity in Crop Breeding

As described by the adage “health comes from the farm, not the pharmacy,” crops serve as sources of metabolites essential for the nutrition and health of humans. Ongoing international biofortification research and breeding programs strive to improve life and well-being (Riezzo et al., 2005). Vitamins and *anthocyanins* are important targets for biofortification because they are sourced primarily from food.

### Vitamins

Structural genes and their origination are essential for biofortification. A group of fat-soluble C20 carotenoid derivatives are denoted as vitamin A, including retinal, retinol and its esters, and retinoic acid. Certain carotenoids, referred to as provitamin A, are cleaved to form vitamin A within the body (Yeum and Russell, 2002). Vitamin A is essential for human health and development (West et al., 2002). Golden rice was developed to deliver provitamin A to ease the global deficiency of vitamin A; in this rice, the carotenoid biosynthetic pathway is reconstituted in the endosperm. Although the endosperm of rice cultivars does not accumulate provitamin A, the earlier intermediate geranylgeranyl diphosphate is present in rice endosperm, which can produce the phytoene under the catalization of plant phytoene synthase (PSY). A transgenic approach was adopted to accumulate provitamin A in the endosperm of rice by expressing a *PSY* and a bacterial phytoene desaturase (*CrtI*) (Ye et al., 2000). Genetically modified golden rice produces as much as 1.6 mg/g total carotenoids in the endosperm, leading to its characteristic yellow color. The limiting and major regulatory step for carotenoid biosynthesis is thought to be phytoene synthase (Fraser et al., 1994; Ronen et al., 1999; Fraser et al., 2002). To increase the carotenoid content of golden rice, systematic tests of psy genes from different plant species were carried out. Hence, “Golden Rice 2” was created by expressing the maize-originated psy gene and the *CrtI* gene from *Erwinia uredovora*, leading to the accumulation of up to 37 ug/g total carotenoids in the endosperm and preferential production of β-carotene (Paine et al., 2005).

Biofortification can be carried out by ectopic expression of metabolic pathways in crops. Astaxanthin, a red ketocarotenoid synthesized from β-carotene, is used in feedstuffs as a supplement. BKT and β-carotene hydroxylase are essential for the producing of astaxanthin (Higuera-Ciapara et al., 2006). Although different hydroxylated carotenoids pile up in the majority of higher plants, the biosynthesis of ketocarotenoids is impaired due to the absence of BKT genes (Cunningham and Gantt, 2005; Zhu et al., 2009). Astaxanthin has been successfully ectopically expressed in several species with the presence of native β-carotene by introducing two (β-carotene hydroxylase and BKT) transgenes or a single (BKT) transgene (Hasunuma et al., 2008; Jayaraj et al., 2008; Huang et al., 2013; Harada et al., 2014; Campbell et al., 2015; Farre et al., 2016). However, rice endosperm does not accumulate β-carotene, which can be preferentially produced by overexpressing *ZmPSY1* and *PaCrtl* (Paine et al., 2005). Zhu et al. developed canthaxanthin rice, which has a high ketocarotenoid content, by expressing *ZmPSY1*, *PaCrtl*, and *CrBKT* in the rice endosperm (**Figure 1**) (Zhu et al., 2018b).

Folate, only synthesized *de novo* in plants and microorganisms, decreases the risk of several diseases (Iyer and Tomar, 2009). Folate biofortification was performed in both tomato fruits and rice seeds (Diaz De La Garza et al., 2007; Storozhenko et al., 2007). To increase the folate content in tomato and rice, two genes encoding enzymes in folate biosynthesis were overexpressed, including an aminodeoxychorismate synthase and a GTP cyclohydrolase I. However, folate is prone to degradation upon storage (Blancquaert et al., 2015) since it is sensitive to oxidation, pondus hydrogenii, and temperature (Scott et al., 2000; De Brouwer et al., 2007). Approximately 40% of the folate remained in the rice grain after storage for 8 months, indicating that the folate may be protected from degradation since folate polyglutamylation by folylpolyglutamate synthetase (FPGS) or complexation with folate-binding proteins (FBP) can improve folate stability (Blancquaert et al., 2010; Blancquaert et al., 2014). Blancquaert et al. aimed to enhance folate concentration and stability by overexpressing the *A. thaliana* cDNA encoding FPGS (ctF) or a synthetic soluble FBP. The transgenic rice supplies 150 times more folate than the wild type (Blancquaert et al., 2015).

### Anthocyanins

Transcriptional regulation of the genes in entire metabolic pathways provide effective tools for metabolic engineering. Anthocyanins are viewed as compounds beneficial for human health, which decrease the risk of certain cancers and other diseases (Wang and Stoner, 2008; Deng et al., 2013; Zhang et al., 2014). Butelli et al. overexpressed the Delila (*Del*) and Rosea1 (*Ros1*) genes from the snapdragon *Antirrhinum majus* in tomato, which encode a basic helix-loop-helix transcription factor and a MYB-related transcription factor, respectively. The transgenic tomatoes exhibited significantly activated transcription levels of key genes, including almost all of the genes required for anthocyanin biosynthesis and genes essential for side-chain modification. Consequently, overexpression of Del/Ros1 activated the production of anthocyanins in tomatoes, resulting in a purple color (Butelli et al., 2008). AtMYB12 driven by the fruit-specific E8 promoter increases the expression levels of genes in primary metabolism and flavonol and hydroxycinnamic ester biosynthesis in tomato and activates the accumulation of both flavonols and caffeoyl quinic acids. Indigo tomato was developed by crossing AtMYB12 tomato with the purple Del/Ros1 tomato line, which accumulates even greater amounts of chlorogenic acid, flavonols, and anthocyanins (Zhang et al., 2015). Although anthocyanins accumulate in several tissues of plants, the endosperm of cereals lacks anthocyanins. The pericarp of some special varieties of rice accumulates anthocyanins and proanthocyanidins. Many efforts have been made to decode the sophisticated anthocyanin biosynthesis pathway in plants, leading to the identification of conserved enzymes, as well as several regulatory proteins (Hichri et al., 2011; Dixon et al., 2013; Zhang et al., 2014; Yuan and Grotewold, 2015). To develop rice with a high anthocyanin content in the endosperm, eight anthocyanin pathway genes were transferred into rice calli, including six structural genes for anthocyanin biosynthesis from Coleus and two regulatory genes. The transgenic plants displayed purple endosperm due to the activated accumulation of anthocyanins and were renamed Zijingmi in Chinese (Zhu et al., 2017).

## Future Perspectives

**Figure 2:**
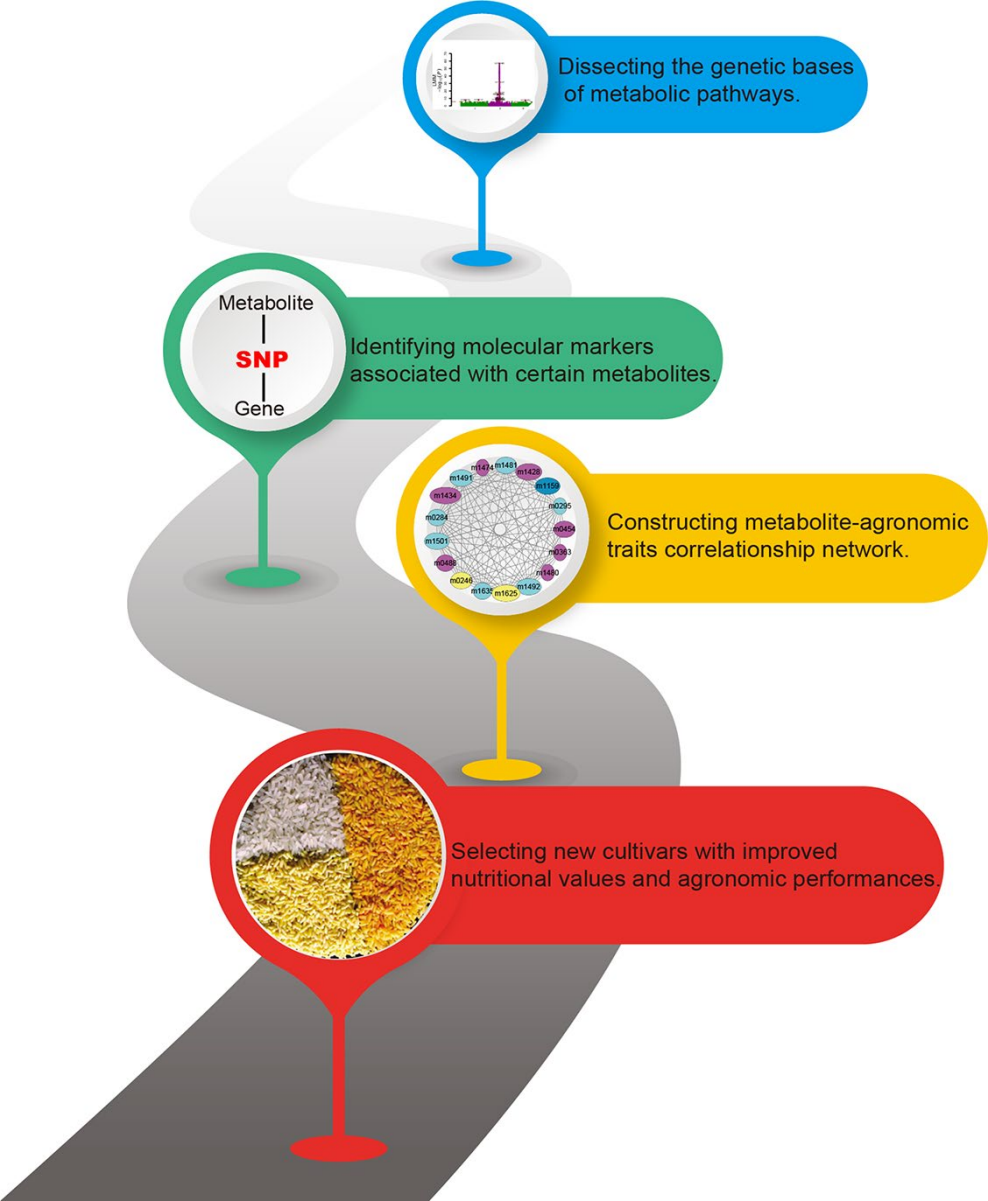
Contributions of metabolomics for metabolome-assisted breeding. This flow chart shows how the metabolome can be used to guide the improved quality of crops. Multi-omics integration analysis is used to analyze the genetic basis of crop nutrients, to explore molecular markers that determine the content of nutrient metabolites, and to establish an interaction network of metabolites, markers, genes, and important agronomic traits to guide the precise breeding of metabolome assisted. The figure is modified with permission from Elsevier (Al-Babili and Beyer, 2005).

Past research has focused largely on annotating more metabolites and decoding metabolic pathways. However, a far more exciting research front in crop breeding has been produced by the multi-omics studies. Here, we have reviewed recent advances, focusing on the diversity of phytonutrients and its genetic bases. Our knowledge on the biosynthesis and the diversity of plant metabolites will be enhanced by studies with multi-omics data. The metabolome-assistant breeding will contribute greatly to the improvement of crops with additional nutritional value (**Figure 2**) and that natural and artificial populations of crops will provide vast gene resources and parental materials.

## Author Contributions

SW and CF wrote the manuscript. SW, CF and JL revised the manuscript.

## Funding

The research conducted in our laboratories was supported by Hainan Provincial Natural Science Foundation of China (319QN155), the National Science Fund for Distinguished Young Scholars (31625021), the State Key Program of the National Natural Science Foundation of China (31530052), the National Natural Science Foundation of China (31800250), and the Hainan University Startup Fund (KYQD(ZR)1866 to JL, KYQD(ZR)1824 to CF, KYQD(ZR)1916 to SW).

## Conflict of Interest Statement

The authors declare that the research was conducted in the absence of any commercial or financial relationships that could be construed as a potential conflict of interest.
